# Toward a Quantitative Estimate of Future Heat Wave Mortality under Global Climate Change

**DOI:** 10.1289/ehp.1002430

**Published:** 2010-12-30

**Authors:** Roger D. Peng, Jennifer F. Bobb, Claudia Tebaldi, Larry McDaniel, Michelle L. Bell, Francesca Dominici

**Affiliations:** 1 Department of Biostatistics, Johns Hopkins Bloomberg School of Public Health, Baltimore, Maryland, USA; 2 Climate Central, One Palmer Square, Princeton, New Jersey, USA; 3 Department of Statistics, University of British Columbia, Vancouver, British Columbia, Canada; 4 National Center for Atmospheric Research, Boulder, Colorado, USA; 5 School of Forestry and Environmental Studies, Yale University, New Haven, Connecticut, USA; 6 Department of Biostatistics, Harvard School of Public Health, Boston, Massachusetts, USA

**Keywords:** climate models, extreme weather events, global warming, population health, time-series models

## Abstract

**Background:**

Climate change is anticipated to affect human health by changing the distribution of known risk factors. Heat waves have had debilitating effects on human mortality, and global climate models predict an increase in the frequency and severity of heat waves. The extent to which climate change will harm human health through changes in the distribution of heat waves and the sources of uncertainty in estimating these effects have not been studied extensively.

**Objectives:**

We estimated the future excess mortality attributable to heat waves under global climate change for a major U.S. city.

**Methods:**

We used a database comprising daily data from 1987 through 2005 on mortality from all nonaccidental causes, ambient levels of particulate matter and ozone, temperature, and dew point temperature for the city of Chicago, Illinois. We estimated the associations between heat waves and mortality in Chicago using Poisson regression models.

**Results:**

Under three different climate change scenarios for 2081–2100 and in the absence of adaptation, the city of Chicago could experience between 166 and 2,217 excess deaths per year attributable to heat waves, based on estimates from seven global climate models. We noted considerable variability in the projections of annual heat wave mortality; the largest source of variation was the choice of climate model.

**Conclusions:**

The impact of future heat waves on human health will likely be profound, and significant gains can be expected by lowering future carbon dioxide emissions.

Evidence of climate change attributable to human causes over the past 50 years has been well documented, and the potential impacts on environmental and ecological outcomes has been studied extensively [Intergovernmental Panel on Climate Change (IPCC) 2007]. The effects of climate change on human health are not as well understood but are thought to result from changes in the distribution of various risk factors such as heat waves, floods, droughts, air pollution, aeroallergens, and vector-borne diseases (Ebi et al. 2006; Haines and Patz 2004; Shuman 2010). An important aspect of understanding the overall impact of climate change on human health is how heat waves will affect mortality and morbidity in the future (O’Neill and Ebi 2009; Portier et al. 2010). In the present day, heat waves contribute significantly to mortality. For instance, in the summer of 1995, the city of Chicago experienced a devastating heat wave that was responsible for > 700 excess deaths in a 1-week period (Whitman et al. 1997). Under any scenario of increasing greenhouse gas concentrations, the most robust signals of future climate changes are more severe heat-related extremes, such as increases in the length, frequency, and intensity of heat waves during the course of the current century (Meehl et al. 2007b; Meehl and Tebaldi 2004; Stocker et al. 1992; Stocker and Raible 2005; Tebaldi et al. 2006). Although present-day health effects of hot temperatures have been fairly well characterized (Anderson and Bell 2009; Braga et al. 2001; D’Ippoliti et al. 2010; Nicholls 2009; O’Neill et al. 2003), the extent to which future changes in the heat wave distribution will affect human health has not been studied as extensively.

Our goal in the current study was to quantify the excess mortality associated with heat waves in Chicago, Illinois, for the years 2081–2100 under several global climate change scenarios. We chose Chicago because of its history of heat waves and because it is a major metropolitan area in the United States. An important aspect of this analysis was the partitioning of uncertainty in the estimation of heat wave health effects. Although there are numerous important sources of uncertainty, we focused on uncertainty due to statistical variation, climate models, and climate change scenarios.

## Materials and Methods

### Data

We obtained the data for this study from the National Morbidity, Mortality, and Air Pollution Study (NMMAPS) database (Samet et al. 2000). The NMMAPS currently contains daily time-series data on mortality, weather, and air pollution that were assembled from publicly available sources in 108 cities in the United States from 1987 through 2005. Cause-specific mortality data, aggregated to the level of a city, were obtained from the National Center for Health Statistics. In each of the 108 cities, daily death counts were available, except for accidental deaths and the deaths of nonresidents who died in the city during the time the data were collected. We used death certificates to calculate daily all-cause mortality by summing the deaths for each day.

Hourly temperature and dew point temperature for the city were obtained from the National Climatic Data Center (2006). The maximum 24-hr temperature was computed for each day in the time period. If more than one monitor was available, the maximum of the maxima from each monitor was used as the final temperature level. Air pollution data for ozone were obtained from the U.S. Environmental Protection Agency Air Quality System (2008) for each city. We used 24-hr integrated average air pollution concentrations, which were measured daily in Chicago. To protect against outliers, a 10% trimmed mean of pollutant values was used to average across monitors in the city after correction for yearly averages for each monitor.

**Figure 1:**
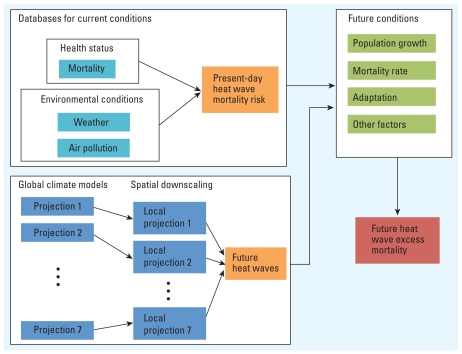
Schematic describing integration of historical mortality, weather, and air pollution data with climate model output to estimate future heat wave excess mortality. The dots are vertical ellipses indicating where other projections would go.

Our approach to estimating future heat wave deaths is depicted in Figure 1. We assembled and linked 19 years (1987–2005) of historical data on daily mortality from all causes (excluding accidents), temperature, and air pollution for the Chicago metropolitan area. Our data set is a time-series of daily weather and mortality data for Chicago, 1987–2005, for three age categories: < 65 years of age, 65–74 years of age, and ≥ 75 years of age. The primary outcome of interest is total nonaccidental mortality. Because the quantity we were interested in obtaining is the relative risk of mortality on a heat wave day versus that same day if it was not part of a heat wave, we considered only those days that have a potential to be heat wave days. Thus, we restricted our analysis to days in the summer season (May–October).

### Heat wave definition

No universally accepted definition of a heat wave is currently available, but most incorporate notions of intense heat experienced over a period of days (Weisskopf et al. 2002). For the purpose of classifying heat waves from temperature data, we used the definition of Meehl and Tebaldi (Huth et al. 2000; Meehl and Tebaldi 2004), acknowledging that estimates of heat wave health effects will necessarily vary with the definition used. The heat wave definition used here relies on two thresholds for daily maximum temperature. Threshold 1 (*T*_1_) is defined as the 97.5th percentile of the distribution of daily maximum temperatures, and threshold 2 (*T*_2_) is defined as the 81st percentile of daily maximum temperatures. A heat wave is then defined as the longest period of consecutive days satisfying the following conditions: the daily maximum temperature is above *T*_1_ for at least 3 days, the daily maximum temperature is above *T*_2_ for every day of the entire period, and the average of daily maximum temperature over the entire period is above *T*_1_. For the maximum temperature data, if values from multiple monitors were available, we used the maximum over all available monitor values as representing the daily maximum for the city.

### Heat wave mortality risk estimation

In the first stage of our approach, we estimated the present-day mortality risk from heat waves using historical data. We considered the following family of log-linear generalized additive models (Hastie and Tibshirani 1990), where *Y_t_* is the number of deaths on day *t* in Chicago and E[*Y_t_*] is the expected mortality:


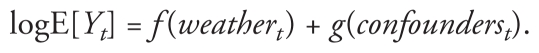


We modeled *Y_t_* to be a member of the quasi-Poisson family to allow for overdispersion in the mortality counts; *f*(•) and *g*(•) are smooth functions modeled using thin-plate splines. The smooth functions remove any medium- to long-term fluctuations in the data but leave the short-term fluctuations needed to estimate the effects of heat waves. Spline structures other than thin-plate splines would be appropriate, but we have conducted extensive sensitivity analyses with respect to the different types of splines and have found that relative risk estimates in time-series models are generally robust to the type of spline used (Peng et al. 2006).

Here, weather variables may include one or more of the following covariates: current-day maximum temperature, average maximum daily temperature of previous 3 days, and current-day 24-hr average dew point temperature. The potential confounding variables we accounted for in the *g*(•) function were current-day 24-hr average ozone levels and smooth temporal fluctuations in time. We also stratified our analysis by three age groups (< 65 years of age, 65–74 years of age, and ≥ 75 years of age) and therefore included intercepts for each age category (< 65 being the baseline category) and interactions of the weather variables with age group in the model. Interactions with age groups were needed because of the differing temporal trends in mortality by age group. The final model was of the form


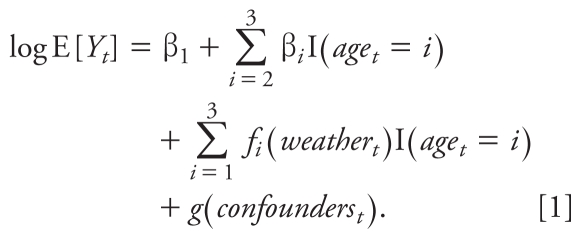


We applied this full model of the weather–mortality relationship to the summer season (May–October), to estimate the relative risk of mortality comparing periods of heat waves with those without heat waves in Chicago for 1987–2005. We fit several models of the form of Equation 1, where the models differed based on which combination of weather covariates were included. For each model, we computed the generalized cross-validation (GCV) criterion (Gu 2002), which evaluates the predictive ability of each model. In the final analysis, we chose the model that minimized the GCV criterion. Using quasi-likelihood procedures (McCullagh and Nelder 1989), we obtained *f*^^^*_i_*, the estimate of the exposure–response function for weather and mortality.

We computed an overall heat wave relative risk for the period 1987–2005 (pooled across the three age groups) by averaging the weather-attributable mortality for heat wave days and dividing by the average weather-attributable mortality for non–heat wave days. Given our log-linear generalized additive model, the relative risk was estimated by


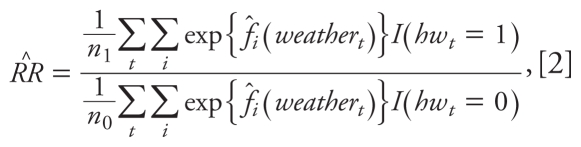


where *i* indexes the three age groups, *hw_t_* is an indicator time series equal to 1 for a heat wave day and 0 otherwise, *n*_1_ is the number of heat wave days, *n*_0_ is the number of non–heat wave days in Chicago during this period, and *I*(•) is an indicator function. We calculated variances and asymptotic 95% confidence intervals (CIs) for the relative risk estimate by applying the delta method (van der Vaart 1998). In addition to computing the overall relative risk from heat waves, we also estimated separate age category-specific relative risks for each of the three age categories separately.

### Projection of future heat wave mortality

In the second stage of our approach, we obtained estimates of future heat waves from seven different climate model simulations of temperature from the Program for Climate Model Diagnosis and Intercomparison (PCMDI 2009) as part of the Coupled Model Intercomparison Project (CMIP3) (Meehl et al. 2007a). (See Table 1 for the complete names of the climate models used.) Heat wave summary statistics for the baseline period 1981–2000 and the future period 2081–2100 period were calculated using the CMIP3 multimodel daily maximum temperature output under the B1, A1B, and A2 scenarios of the IPCC Special Report on Emissions Scenarios (SRES) (IPCC 2000).

The SRES consist of divergent storylines that describe the demographic, economic, and technological changes in the future world. The SRES A1 family of scenarios assumes rapid economic growth, an increase in world population until mid-century followed by a decrease, and the introduction of more-efficient energy sources and conversion technologies. The A1B scenario, in particular, assumes a mix of energy sources that is balanced across fossil fuel and alternative sources. The B1 scenario assumes a highly convergent world with moderate population growth (as with A1), a reduction in material intensity, and the introduction of clean and resource-efficient technologies. The A2 scenario assumes a very heterogeneous world with little convergence between nations, regionally oriented economic development, and continuously increasing global population (IPCC 2000).

For each climate model and SRES combination, the projected change in heat wave frequency and length based on daily maximum temperature at the grid cell covering Chicago was analyzed for the 20-year period 2081–2100 compared with present day. The change in heat wave statistics was calculated relative to the climate model baseline period of 1981–2000. Climate model baseline data were not available for the period 1987–2005 corresponding to our observed data, but the climate model baseline period has substantial overlap with our observed historical data time period. The typical grid cell size for each climate model was on the order of 200 km in both horizontal directions.

Using the daily maximum temperature output from each of the climate models, we calculated the number of heat waves per year and the length of each heat wave (in days) under a changing climate. Averaging each of these numbers over the length of each study period (current and future) and multiplying them together provided the expected number of heat wave days per year for each set of climate change scenarios and climate models, from which we can compute the statistics of change.

Rather than using the number and the length of future heat waves computed by applying our heat wave definition to the climate model temperature output, we calculated the change in the number and length as the ratio between future value and present-day value, as both were simulated from the climate model. The ratio was then multiplied by the present-day number and length of heat waves, as indicated by the observed data, to obtain an estimate of future heat wave characteristics. The use of the ratio between future and present-day values, rather than the absolute difference, minimizes the limitations of the climate models, such as their documented shortcomings in reproducing blocking effects in the atmosphere.

The expected number of excess deaths during a given heat wave period was calculated as


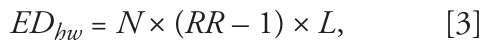


where *N* is the expected daily number of deaths on a non–heat wave day, *L* is the length of the heat wave period in days, and *RR* is the heat wave relative risk. *N* was estimated by calculating the mean daily mortality across all non–heat wave days in the 1987–2005 period. To quantify the overall health impact of heat waves, we computed the annual excess mortality attributable to heat waves, which is the expected number of deaths in a 1-year period caused by all heat waves in that year. This summary of health impact incorporates the change in both the rate at which heat waves occur and the length of heat waves in the future. We calculated this summary by computing for every heat wave in the respective time period (1987–2005 for present day and 2081–2100 for the future period), summing the excess deaths across all heat waves and dividing by the total number of years.

To estimate future excess mortality, we assumed the same non–heat wave rate of mortality as the 1987–2005 period and projected population growth using the B1, A1, and A2 age-stratified population estimates from the International Institute for Applied Systems Analysis (IIASA) for the 2081–2100 period (Lutz 1996). Under all three SRES, the IIASA population growth estimates for North America all project that the 65–74 years of age and ≥ 75 years of age categories will substantially increase in size relative to the < 65 years of age population. When computing the future excess mortality attributable to heat waves, we take into account the changing age structure of the population by applying age category–specific relative risks estimated from the age-stratified time-series models.

## Results

For the 19-year period 1987–2005 in Chicago, there were a total of 14 heat waves (0.7 heat waves per year), and each heat wave lasted 9.2 days, on average. The average daily number of deaths on non–heat wave days for the May–October period was *n* = 102 deaths per day. The overall present-day heat wave relative risk of mortality was estimated from the observed data to be a 7.8% (95% CI, 6.1–9.5) increase in daily mortality during heat waves compared with otherwise similar non–heat wave periods. For the city of Chicago, this relative risk translated to a total of 1,007 (95% CI, 798–1,235) excess deaths across the 19-year period 1987–2005, or an annual excess mortality attributable to heat waves of 53 (95% CI, 42–65) deaths per year. For the age category–specific models, we estimated the relative risk to be an increase of 8.5% (95% CI, 5.9–11.2), 11.0% (95% CI, 7.8–14.2), and 3.5% (95% CI, 1.4–5.5) in daily mortality for the < 65 years, 65–74 years, and ≥ 75 years age categories, respectively.

We chose seven different climate models for which simulations for the three SRES could be obtained (Table 1). From the climate model output, we obtained the annual rate and average length of heat waves for the baseline period 1981–2000 and the future period 2081–2100. Using the data from these two periods, we calculated the change in frequency and length of heat waves across the two periods as predicted by the climate models. This change in heat wave characteristics between the two periods is used to project heat wave mortality into the future period.

Across all three SRES, the climate models projected an annual rate of heat waves in the future (2081–2100) ranging from 0.6 to 5.4 heat waves per year (Table 2). The average lengths of these future heat waves ranged from 6.2 days with the Canadian Centre for Climate Modeling and Analysis model under the B1 scenario to 31.1 days with the Geophysical Fluid Dynamics Laboratory model under the A2 scenario (Table 3). According to the climate model output, from the present day (1981–2000) to the future period (2081–2100) and across the different climate models, the annual number of heat waves increased by a factor ranging from 1.1 to 31.7, while the average length of heat waves increased by a factor ranging from 1.0 to 3.9. Of the 21 climate model/SRES combinations (seven climate models times three SRES), all but one of the combinations projected that the rate of occurrence and the length of heat waves will increase.

**Figure 2:**
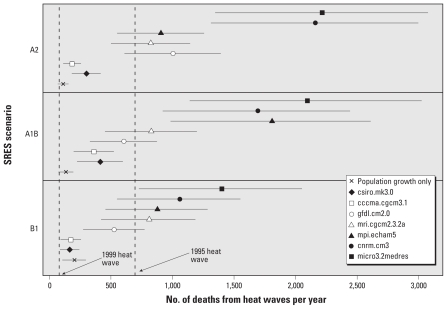
Annual excess mortality attributable to heat waves in Chicago, 2081–2100, for seven climate models under the B1, A1B, and A2 SRES (with 95% CIs reflecting statistical uncertainty in risk estimation). Full names of climate models are provided in Table 1.

Applying the present-day heat wave risk for Chicago to the estimates of heat waves under future conditions, we estimated an annual excess mortality attributable to heat waves ranging between 166 to 2,217 deaths per year (Figure 2). Included in Figure 2 are the excess mortality estimates for the 1995 and 1999 heat waves in Chicago (Naughton et al. 2002; Whitman et al. 1997). Under the A2 scenario, results from five of the seven climate models project that the annual mortality from heat waves will be similar to or greater than the mortality from the devastating 1995 heat wave. All of the climate models under all three scenarios induce projections of the annual heat wave mortality greater than the 1999 heat wave.

As a reference for comparison, in Figure 2 we also projected the change in heat wave mortality in the case where the population increases as predicted for each SRES but there are no effects of climate change on the characteristics of heat waves (indicated in Figure 2 as population growth only). For all but 3 of the 21 projections in Figure 2, the change in heat wave mortality in the future period cannot be attributed solely to the increase in population.

Projections of future heat wave mortality varied considerably across the climate models and across SRES within a climate model. The A1B scenario generally produced the highest mortality estimate for each of the climate models, and the B1 scenario always produced the lowest estimate. Although statistical variation arising from uncertainty about the present-day heat wave relative risk was certainly a factor, most of the variability in mortality projections could be attributed to the choice of climate models and SRES scenarios. An analysis of variance indicated that the choice of climate model explained 81% of the variation in the mortality projections, while the choice of SRES explained another 8%.

## Discussion

In this study, we have estimated future annual excess mortality attributable to heat waves for Chicago, Illinois, a major U.S. city, using several global climate models and climate change scenarios. We found considerable variability in the projections of annual heat wave mortality, with point estimates ranging from 166 to 2,217 deaths per year. In particular, the largest source of variation appeared to be the different climate model implementations, followed by variation due to statistical noise and the choice of SRES. Nevertheless, even in the presence of large intermodel variations, the results of our analysis suggest that annual heat wave mortality will increase in the future and that a mitigation of this projected increase may be expected through a lower pathway of future CO_2_ emissions.

We computed statistics of change in heat waves directly from global climate models, as these models are the most direct source of future climate change projections. We chose not to pursue a more sophisticated downscaling approach to avoid introducing another source of uncertainty and to focus on the variation captured by a range of global circulation models (GCMs). Such a range of results would not have been available as daily output in a downscaled format. Furthermore, changes in temperature fields are relatively smooth in space (particularly over a flat domain like the Chicago area) and, as discussed previously, we focused on relative changes with respect to climatology that should diminish the effect of limitations in the output of the models. In addition, given the large size of the intermodel variability that our study documents, any higher resolution information from a particular climate model would be eclipsed in the range of uncertainty produced by the ensemble analysis.

The methodology outlined here used publicly available data on mortality, weather, and air pollution to estimate the historical and future impact of heat waves on human health and is broadly applicable to estimating future heat wave mortality for locations around the world and to estimating the impacts of other climate-related risk factors such as floods, droughts, and air pollution exposure. A key advantage of our approach is that it can be easily modified with respect to the various inputs and assumptions about the future to obtain predictions from a wide range of plausible scenarios.

Methods for estimating future mortality effects of heat waves necessarily rely on numerous assumptions. We used multiple global climate model simulations of future changes to account for variation among climate models’ structural assumptions, which are recognized to contribute an important source of uncertainty in future projections (Tebaldi and Knutti 2007). Intermodel variability is significant even at global average scales, but it becomes increasingly relevant as the output of global models is used to describe climate change at small regional scales and for high frequency quantities like daily output, as in the case of our analysis. Accordingly, the modeling community has undertaken concerted efforts in performing standard (comparable) simulations and making multimodel output available in publicly accessible archives like PCMDI’s CMIP3 and soon to come CMIP5 (PCMDI 2009). IPCC Working Group 1 uses multimodel ensembles for assessment of future projections, and impact analysis is moving consistently toward considering multiple models, exploring the sensitivity of results to their alternative choices (Knutti et al. 2010a). There is little agreement on how to synthesize different projections from multiple models (Knutti 2010b), however, and even less agreement on how to merge results from different scenarios (Grüber and Nakicenovic 2001; Schneider 2001). Accordingly, our analysis presents the whole range of individual outcomes without trying to achieve a consensus estimate.

This study did not investigate whether some deaths would have occurred only a few days later without the elevated exposures, a concept known as “mortality displacement.” Earlier work on this topic in the context of heat-related mortality found no evidence that short-term mortality displacement explained heat-related mortality for the 2004 heat wave in Brisbane, Australia (Tong et al. 2010) or in a study of 15 European cities for 1990–2000 (Analitis et al. 2008). Associations between high temperatures and mortality for an elderly population in Sweden were robust to adjustment for mortality displacement (Rocklöv and Forsberg 2010). However, evidence of some mortality displacement for heat-related deaths was observed in 15 European cities (Baccini et al. 2008). Approximately 26% of heat-related deaths were due to mortality displacement in a study of the 1995 Chicago heat wave (Kaiser et al. 2007). Further, research based on London, Delhi, and São Paulo found some evidence for mortality displacement in London, but not in Delhi, indicating that regional variation may exist (Hajat et al. 2005).

Knowlton et al. (2007) used methods similar to those used in this study and applied them to data from New York City to project heat-related excess mortality. That study used a single GCM as well as the A2 and B2 SRES to project a 65–295% increase in excess mortality in 2050; this increase was reduced when acclimatization was taken into account. A study of six cities in the United States, Europe, and Australia found that both the shift in mean temperature and the change in temperature variability in the future can contribute separately to changes in heat-related mortality (Gosling et al. 2009). A study of three Canadian cities found that in 2080 there would be significantly increased mortality in summer along with a slight decrease in winter (Doyon et al. 2008). They found that differences in mortality projections between SRES were not significant. In each of these three studies, a single GCM was used to project future climate conditions.

We acknowledge that this does not represent a comprehensive evaluation of modeling uncertainties, even conditionally on the specific scenario used. Rather, we propose this as a first-order quantification of this source of variation. If anything, more extensive explorations of modeling uncertainties seem to indicate that these models provide a conservative estimate of the potential changes (Tebaldi and Knutti 2007). For example, one aspect of present and future heat waves that we did not explore here is the intensity of each heat wave (i.e., the magnitude of the temperature during a heat wave), which is also expected to increase in the future (Meehl and Tebaldi 2004). Given the positive heat wave risk estimated here, any increase in the intensity of heat waves in the future would likely increase our estimates of excess mortality.

We also used climate projections under three different SRES that describe very different future global climate regimes. The SRES cover a wide range of possibilities with respect to economic development, future CO levels, and technological contributions. Although the IPCC does not specifically place probabilities on the likelihood of each scenario occurring, our estimation of future heat wave mortality under each of these scenarios allows us to systematically assess the variability introduced by the different possible scenarios.

Although we have attempted to address some sources of uncertainty in this analysis, our results still necessitate several assumptions. Our results assume that the baseline rate of mortality on non–heat wave days is the same in the future as it is for the present day. The estimates also assume that there is no adaptation to extreme heat, so that the mortality risk from heat waves is constant over time. These assumptions are likely oversimplifications given recent trends in mortality rates and in the adoption of air conditioning (Rogot et al. 1992). For example, the presence of central air conditioning in Chicago housing units has risen steadily for 1995–2003 from 47% of all housing units to 60% (U.S. Census Bureau 2004). In our analysis, we do not adjust for air conditioning use, early warning systems, and other factors that could lower the mortality impact of heat waves under a changing climate. Further, additional climate change scenarios with more or less stringent control of greenhouse gases could be explored, as well as more definitions of heat waves. In the next few years, new scenarios at higher resolution from both global climate models and regional climate models will become available and are expected to represent more accurately local climate change effects (such as blocking effects) that are relevant for extreme heat statistics.

Climate change is anticipated to exacerbate a wide range of human health risks, including impacts from infectious disease, environmental refugees, and air pollution (Patz et al. 2005). This work presents one of the first efforts to quantify the impacts of heat waves under a changing climate on human mortality on a local scale, by coupling global climate change models with data on air pollution, weather, and human health. Our approach could be easily modified with respect to various inputs and assumptions about the future to obtain predictions from a wide range of climate-change scenarios. Given our results concerning the variability of mortality estimates across climate model implementations, future studies should carefully consider this source of uncertainty in making projections of the future health burden of climate change.

## Figures and Tables

**Table 1:** Climate models used in projections of future temperature.

Climate model	Originating group
cccma.cgcm3.1	Canadian Centre for Climate Modeling and Analysis
cnrm.cm3	Météo-France/Centre National de Recherches Météorologiques
csiro.mk3.0	CSIRO Atmospheric Research (Australia)
gfdl.cm2.0	Geophysical Fluid Dynamics Laboratory/NOAA (USA)
miroc3.2.medres	Center for Climate System Research/JAMSTEC (Japan)
mpi.echam5	Max Planck Institute for Meteorology (Germany)
mri.cgcm2.3.2a	Meteorological Research Institute (Japan)

**Table 2:** Annual number of heat waves predicted by each climate model and SRES scenario combination for the model grid cell containing Chicago in the present-day period 1981–2000 and the future period 2081–2100.

	SRES scenario					
	1981–2000^a^			2081–2100		
Climate model	B1	A1B	A2	B1	A1B	A2
cccma.cgcm3.1	0.30	1.20	0.30	0.65	1.40	1.05
cnrm.cm3	0.30	3.00	0.30	1.80	3.30	4.00
csiro.mk3.0	0.20	1.00	0.20	0.60	1.45	1.05
gfdl.cm2.0	0.45	1.30	0.45	1.15	2.10	1.70
miroc3.2.medres	0.15	1.00	0.15	3.20	5.40	4.75
mpi.echam5	0.40	1.10	0.40	2.65	5.20	3.95
mri.cgcm2.3.2a	0.20	1.00	0.20	1.70	2.55	2.95

The SRES A1B family of scenarios assumes rapid economic growth, an increase in world population until mid-century followed by a decrease, and the introduction of more efficient energy sources and conversion technologies where the mix of energy sources is balanced across fossil fuel and alternative sources. The B1 scenario assumes a highly convergent world with moderate population growth (as with A1B), a reduction in material intensity, and the introduction of clean and resource-efficient technologies. The A2 scenario assumes a very heterogeneous world with little convergence between nations, regionally oriented economic development, and continuously increasing global population (IPCC 2000). Full names of climate models are provided in Table 1.

a Climate model values for the period 1981–2000 were used to calculate the change in heat wave frequency between the present-day and future periods.

**Table 3:** Average length (in days) of heat waves in 1981–2000 and 2081–2100, predicted by each climate model and SRES scenario combination, for the model grid cell containing Chicago.

	SRES scenario					
	1981–2000^a^			2081–2100		
Climate model	B1	A1B	A2	B1	A1B	A2
cccma.cgcm3.1	6.33	6.33	6.33	6.23	9.00	7.33
cnrm.cm3	5.33	5.33	5.33	11.44	14.98	18.94
csiro.mk3.0	8.25	8.25	8.25	8.33	12.90	15.52
gfdl.cm2.0	8.00	8.00	8.00	13.39	20.86	31.06
miroc3.2.medres	6.00	6.00	6.00	9.58	12.76	18.41
mpi.echam5	5.75	5.75	5.75	6.94	10.96	8.67
mri.cgcm2.3.2a	5.25	5.25	5.25	9.12	9.37	9.64

Full names of climate models are provided in Table 1.

a Climate model values for the period 1981–2000 were used to calculate the change in heat wave length between the present-day and future periods.
